# Development and Application of Analytical Method for Simultaneous Quantification of Iopromide and Iodixanol in Human Plasma

**DOI:** 10.1155/ianc/2432521

**Published:** 2025-12-06

**Authors:** Liya Ye, Nianxi Yu, Peng Huan, Zhengyan Liang, Zhenhui Jiang, Xinran Wang, Zhipeng Wang, Xia Tao, Shouhong Gao

**Affiliations:** ^1^ College of Traditional Chinese Medicine, Yunnan University of Traditional Chinese Medicine, Kunming, Yunnan, China, ynutcm.edu.cn; ^2^ Department of Pharmacy, Second Affiliated Hospital of Naval Medical University, Shanghai, China; ^3^ Dental Clinic of Shanghai Yangpu District, Shanghai, China

**Keywords:** iodixanol, iopromide, therapeutic drug monitoring, UHPLC-MS/MS

## Abstract

The purpose of this study is to develop and validate a simple and rapid analytical method using ultrahigh performance liquid chromatography–tandem mass spectrometry (UHPLC‐MS/MS) for the simultaneous quantification of two commonly used contrast agents, iopromide and iodixanol, in human plasma. The separation of two compounds was analyzed utilizing an Agilent ZORBAX SB‐C_18_ column (2.1 × 100 mm, 3.5 μm) with an isocratic elution procedure. The mobile phase consisted of acetonitrile and 0.2% formic acid aqueous solution (14:86, V:V). A simple protein precipitation method was used to pretreat plasma samples. The iopromide and iodixanol exhibited excellent linearity between 2.0 and 400.0 μg/mL, with both *R* values exceeding 0.99. Recovery of iopromide ranged from 91.39% to 102.69%, and matrix effect varied between 87.88% and 104.08%; the relative standard deviation (RSD%) of intra‐ and interday precisions fell within the range of 1.29%–4.7%. For iodixanol, recovery ranged from 97.68% to 100.14%, and the matrix effect was between 87.88% and 96.64%, and RSD% values of intra‐ and interday precisions ranged from 1.58% to 8.2%. Method validation results all met methodological criteria. The UHPLC‐MS/MS method was successfully developed and validated and then applied to determine two common contrast agents, iopromide and iodixanol, in human plasma.

## 1. Introduction

As is widely recognized in the field of diagnostic imaging, computed tomography (CT) scanning offers several advantages, including high resolution, noninvasiveness, safety, cost‐effectiveness, and speed. Consequently, it has become the preferred diagnostic modality for numerous diseases [1–4]. Continuous advancements in medical imaging technology have significantly enhanced both the diagnosis and treatment of various diseases, thereby improving patient outcomes and quality of life. Nevertheless, when certain lesions are atypical or cannot be distinctly identified on a conventional CT scan, contrast enhancement is often achieved using traditional iodine‐ or barium‐based contrast media (CM) [1, 5]. In clinical CT, conventional CM serves as a powerful diagnostic tool that is widely employed to amplify X‐ray attenuation in vascular structures or target organs such as the urinary tract and gastrointestinal tract. This enhancement improves the contrast of reconstructed images obtained from both static and dynamic CT scans [6–8]. Furthermore, intravenous iodinated contrast agents can improve the accuracy of qualitative disease diagnosis in CT [6, 9, 10]. Currently, iodinated contrast agents (e.g., iopromide and iodixanol) represent the gold standard for intravenous X‐ray contrast agents used in CT or interventional angiography [6].

These iodinated contrast agents exhibit similar efficacy and favorable safety profiles. Although iodine is biologically inert, iodinated contrast agents may still elicit adverse reactions. To effectively mitigate potential adverse effects associated with these CM prior to their administration, it is essential to inquire about patients’ medical histories—specifically regarding diabetes mellitus and renal function—as well as any previous reactions to CM (if applicable) and history of CM‐related allergies [11].

At present, there are limited quantitative studies on intravascular contrast media (ICM) in patients. During the literature review process, it was noted that only Denis et al. established a liquid chromatography–mass spectrometry (LC‐MS) detection method for iohexol and iodixanol in 2007, enrolling 17 patients. This preliminary investigation suggested that the renal clearance rate of iohexol could serve as a potential marker for evaluating renal injury in clinical settings [12]. However, we have not yet found validated analytical methods for the simultaneous quantification of iopromide and iodixanol in human plasma. Previous studies have focused primarily on quantifying either iopromide or iodixanol individually, leaving the simultaneous analysis of both agents—essential for clinical scenarios involving in cross‐evaluation—understudied. Most existing methods are confined to single‐agent detection, whereas our approach addresses clinical situations requiring comparative use of both contrast agents in practice. Notably, iopromide and iodixanol may exhibit pharmacokinetic variability in clinical use—a phenomenon that mirrors observations from studies of other compounds such as kokusaginine, where sex‐specific differences in metabolism and bioavailability markedly influence drug exposure [13]. This underscores the need for robust analytical techniques (e.g., simultaneous quantification) to support personalized pharmacokinetic assessments. We therefore developed a rapid method for the simultaneous quantification of iopromide and iodixanol. Using this method, we analyzed drug concentrations from more than 200 patients and performed a preliminary evaluation of in vivo exposure levels for both agents. Reliable plasma drug quantification methods, such as the one presented here for contrast agents, are indispensable across various therapeutic and research domains. This importance is not limited to CM—for instance, recent research on overactive bladder treatments demonstrates that precise plasma concentration analysis is crucial for optimizing dosing regimens and elucidating therapeutic efficacy [14].

## 2. Materials and Methods

### 2.1. Chemicals and Reagents

The experimental standards used in this study were iopromide (purity ≥ 98%), iodixanol (purity ≥ 98%), and internal standard iopromide‐D_3_ (purity ≥ 97%), all of which were purchased from Shanghai Yuanye Biotechnology Co., Ltd. (Shanghai, China). The organic solvents employed throughout this experiment included methanol (MeOH) and acetonitrile (ACN) from Merck (Darmstadt, Germany), as well as formic acid (FA) obtained from Macklin (Shanghai, China). Distilled water (H_2_O) was obtained from Watsons Distilled Water Co., Ltd. (Shenzhen, China). All other reagents used in the experiments were at least of analytical grade.

### 2.2. Instrumentation

All experiments in this study were conducted using an Agilent 1290 ultrahigh performance liquid chromatography (UHPLC) system coupled to an Agilent 6460A mass spectrometer (MS) system. The instrument system used the Agilent Jet Stream electrospray ionization (AJS‐ESI) source. The software platform utilized the Agilent MassHunter data processing software (Version 6.0.0). Qualitative and quantitative analyses were performed using Agilent Qualitative/Quantitative Analysis B.07.00 software (Agilent, USA).

### 2.3. Chromatographic Conditions

The separation of the compounds was carried out on a ZORBAX SB‐C18 column (2.1 × 100 mm, 3.5 μm, Agilent, MA, USA). The compounds were separated using an isocratic elution program, with the column temperature maintained at 35°C. The mobile phase comprised a mixture of ACN and 0.2% FA aqueous solution (14:86, V:V), enabling the separation of the three compounds within 2.3 min at a flow rate of 0.3 mL/min.

### 2.4. Mass Spectrometry Conditions

Mass spectrometry detection was performed in multireaction monitoring (MRM) mode using an electrospray ionization (ESI) source, with both positive and negative ionization modes employed as appropriate. Nitrogen was used as both the nebulizer gas and drying gas, while high‐purity nitrogen at 0.2 MPa was employed as the collision gas. The MS parameters were set as follows: capillary voltage: 4500 V, nebulizer pressure: 45 psi, dry gas flow rate: 12 L/min, dry gas temperature: 320°C, sheath gas temperature: 300°C, and sheath gas flow rate: 12 L/min. The injection volume was 5 μL, with quantitative analysis based on mass‐to‐charge ratios (m/z) detailed in Table 1.

**Table 1:** Mass spectrometry detection parameters of analytes and internal standard.

Compound	Precursor ion	Product ion	Fragmentor (V)	Collision energy (V)	Polarity
Iodixanol	1551.0	1551.0	180	0	Positive
Iopromide	789.5	126.9	135	12	Negative
Iopromide‐D3 (IS)	792.7	126.9	135	12	Negative

### 2.5. Preparation of Standard and Quality Control (QC) Samples

To prepare standard solutions, 20.0 mg of iodixanol and iopromide standards were precisely weighed separately and placed into 1‐mL volumetric flasks, followed by dissolution in MeOH. The final volume was adjusted to exactly 1 mL, resulting in stock solutions with a concentration of 20 mg/mL. Both stock solutions were aliquoted in small volumes and stored at −80°C for preservation. Stock solutions of iodixanol and iopromide were subsequently added separately to drug‐free human plasma to prepare a series of calibration standards, with concentration of 2, 10, 20 , 40, 50, 80, 100, 200, 320, and 400 μg/mL. The QC samples (low: 20.0 μg/mL, medium: 80.0 μg/mL, and high: 320.0 μg/mL) were prepared separately.

### 2.6. Sample Pretreatment

For plasma sample pretreatment, protein precipitation was performed as follows: 100 μL of plasma sample was first added to a 1.5‐mL centrifuge tube, followed by the addition of 300 μL of precipitant solution (MeOH containing the internal standard at a concentration of 10.0 μg/mL). The mixture was thoroughly vortexed for 3 min and then centrifuged at 12,000 × g for 10 min. Finally, 5 μL of the supernatant was injected into the UHPLC‐MS/MS system for determination.

### 2.7. Methodological Study

Method validation for iodixanol and iopromide in human plasma was conducted in accordance with the *Bioanalytical Method Validation Guidance for Industry* and the *Chinese Pharmacopoeia* (2020 edition) [15, 16]. A comprehensive evaluation was performed covering the following parameters: specificity, linearity, interday/intraday accuracy and precision, extraction recovery, matrix effect, stability, and dilution effect.

Specificity was assessed by analyzing at least six different batches of blank human plasma and blank plasma spiked with the analytes and internal standard; interference was evaluated by comparing chromatographic peaks among the groups. The acceptance criterion for specificity was that interfering peaks in blank plasma did not exceed 15% of the peak area of the analytes at the lower limit of quantification (LLOQ) and did not exceed 5% of the peak area of the internal standard.

Calibration curves for both analytes were constructed using a linear regression model, with the dependent variable as the ratio of the analyte peak area to the internal standard peak area, and the independent variable as the nominal concentration. The LLOQ was defined as the lowest concentration that could be accurately quantified. Linearity was confirmed across three independent batches, with back‐calculated deviations within ±15% for all concentration points (±20% for LLOQ).

For the evaluation of interday and intraday precision and accuracy: Five replicate spiked samples were prepared for each of the three QC levels (low, medium, and high) and the LLOQ level in a single batch (for intraday assessment); this process was repeated across three independent batches (for interday assessment). The acceptance criteria were as follows: intraday and interday RSD% ≤ 15% (≤ 20% for LLOQ), and intraday and interday RE% within ±15% (±20% for LLOQ).

Extraction recoveries and matrix effects were evaluated using QC samples at three concentration levels. Extraction recoveries were calculated by comparing the peak areas of spiked samples to those of spiked postextraction plasma samples at the same concentration levels. Matrix effects were evaluated by comparing the peak areas of spiked postextraction plasma samples to those of standard solutions in solvent at the same concentration levels. The acceptance criterion was RSD% ≤ 15% for both extraction recovery and matrix effect.

Stability assessments were conducted to simulate actual storage conditions during clinical sample handling, including 4‐h stability at room temperature (25°C), 24‐h postprocessing stability in the autosampler, freeze‐thaw stability (three freeze‐thaw cycles), and long‐term stability at −80°C for 30 days.

The dilution effect was assessed by preparing a spiked plasma sample at a concentration higher than the upper limit of the calibration range, diluting it to fall into the calibration range, and comparing the measured concentration of the diluted sample to its nominal concentration. The acceptance criteria were RSD% < 15% and RE% within ±15% for each dilution factor.

## 3. Results and Discussion

### 3.1. Method Development

This study developed an UHPLC‐MS/MS method for the simultaneous quantification of iopromide and iodixanol in human plasma, using protein precipitation with MeOH as the precipitant and Iopromide D3 as the internal standard. The analysis time for a single plasma sample was only 2.3 min, which represents a substantial reduction in both time and solvent usage compared to the earlier method [12]. The method also exhibits a LLOQ of 2 μg/mL, making it applicable for measuring low plasma concentrations at the final sampling time point [12, 17].

### 3.2. Method Validation

#### 3.2.1. Specificity

Chromatogram comparisons between blank plasma and plasma spiked with the analytes (iopromide and iodixanol) and internal standard across six different batches revealed no interfering peaks at the retention times of iopromide, iodixanol, or the internal standard. These chromatograms are presented in Figure 1, demonstrating satisfactory specificity of the method.

Figure 1Comparative MRM chromatograms for specificity. (a) Blank plasma matrix, (b) blank plasma sample spiked with IS, (c) LLOQ sample, and (d) a real sample.

**Figure:**
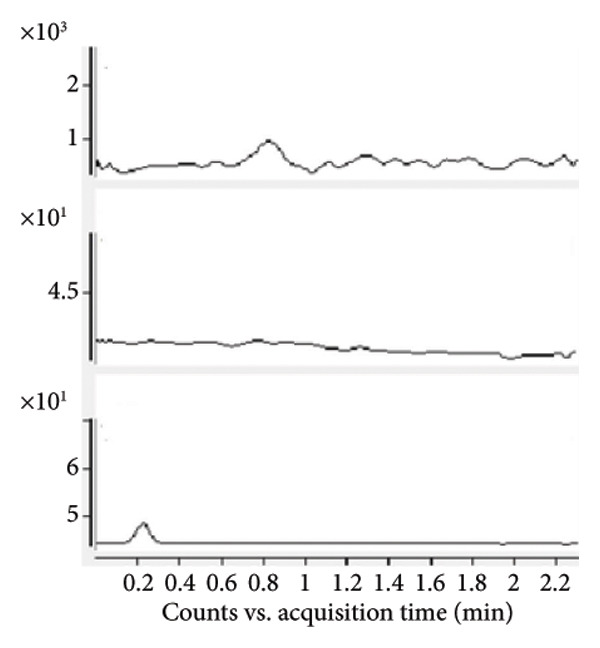
(a)

**Figure:**
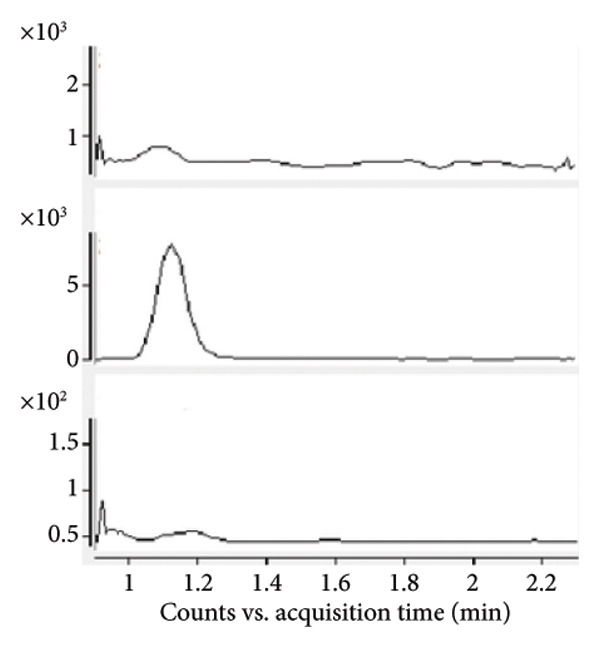
(b)

**Figure:**
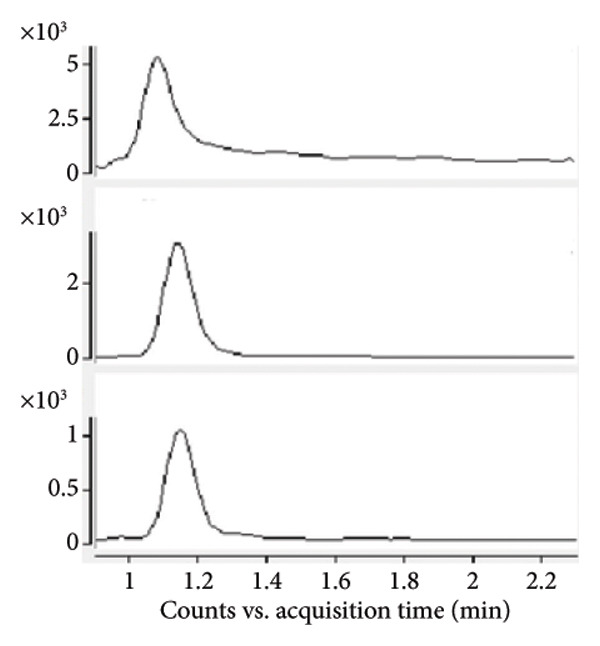
(c)

**Figure:**
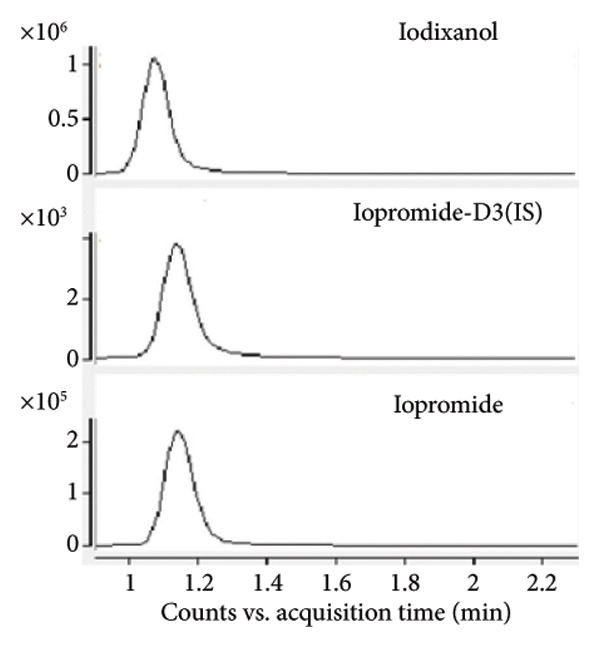
(d)

#### 3.2.2. Linearity of the Calibration Curves and LLOQ

**Figure 2:**
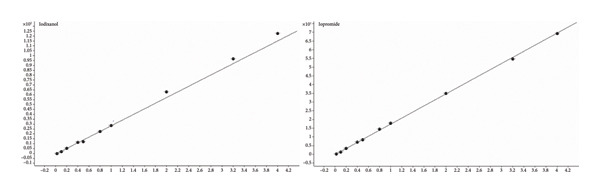
Plots of calibration curves of iopromide and iodixanol in human plasma.

The linear range for both analytes ranged from 2.0 to 400.0 μg/mL. Iopromide exhibited a correlation coefficient (*r*) greater than 0.999, and iodixanol showed a correlation coefficient (*r*) exceeding 0.992 (data are shown in Table 2 and Figure 2). The LLOQs for both iopromide and iodixanol were established at 2.0 μg/mL. To verify the LLOQ performance, six replicate spiked samples at the LLOQ concentration were analyzed over at least 2 days. The RSD% of the LLOQ for both analytes was less than 1.57%, and the RE% was less than 14.8% (data are presented in Table 3), which met the requirements of the Pharmacopoeia (2020 edition).

**Table 2:** Regression parameters of calibration curves for iopromide and iodixanol.

Compound	Linear range (μg/mL)	Calibration curve	Correlation coefficient (*r*)
Iopromide	2.0∼400.0	*y* = 17.407438 ∗ χ − 0.092742	0.9995
Iodixanol	2.0∼400.0	*y* = 28.868482 ∗ *χ* − 0.584515	0.9929

**Table 3:** Assessment of LLOQ of iopromide and iodixanol in human plasma (*n* = 6).

Compound	Nominal concentration (μg/mL)	Measured concentration (mean ± SD, μg/mL)	Precision (RSD%)	Accuracy (RE%)
Iopromide	2.0	2.3 ± 0.04	1.57	14.80
Iodixanol	2.0	2.15 ± 0.02	0.95	7.60

#### 3.2.3. Interday and Intraday Precision and Accuracy

For both iopromide and iodixanol, intraday and interday precision and accuracy were evaluated using three QC concentration levels: low (20.0 μg/mL), medium (80.0 μg/mL), and high (320.0 μg/mL), along with the linear range assessment. Detailed results are presented in Table 4. The results demonstrated that the RSD% of intraday and interday precisions of both analytes were less than 8.2%, and the RE% were less than 13.0%, indicating that the precision and accuracy of the method were acceptable.

**Table 4:** The intraday and interday precision and accuracy of iopromide and iodixanol in human plasma (*n* = 5).

Compound	Nominal concentration (μg/mL)	Intraday			Interday		
		Mean ± SD	RSD%	RE%	Mean ± SD	RSD%	RE%
Iopromide	20.0	19.30 ± 0.25	1.29	3.50	19.92 ± 0.69	3.50	0.40
	80.0	73.06 ± 1.63	2.24	8.60	68.85 ± 3.62	3.50	13.0
	320.0	305.66 ± 6.18	2.02	4.50	321.06 ± 15.08	4.70	0.30

Iodixanol	20.0	20.14 ± 0.58	2.87	0.69	18.49 ± 1.32	7.10	7.50
	80.0	78.67 ± 1.85	2.30	1.60	82.78 ± 6.76	8.20	3.40
	320.0	347.09 ± 5.46	1.58	8.5	342.82 ± 23.44	6.80	7.10

#### 3.2.4. Matrix Effect and Extraction Recovery

Freshly prepared samples at QC concentration levels of iopromide and iodixanol were analyzed for extraction recoveries and matrix effects, confirming the feasibility of the pretreatment method. The extraction recovery for iopromide ranged from 91.39% to 102.69%, while its matrix effect varied between 93.24% and 104.08%. For iodixanol, extraction recovery fell between 97.68% and 100.14%, with matrix effects ranging from 87.88% to 96.64%. All results meet the validation criteria with RSD% less than 5.24%, and detailed information is provided in Table 5.

**Table 5:** Extraction recovery and matrix effect of iopromide and iodixanol in human plasma (*n* = 3).

Compound	Nominal concentration (μg/mL)	Extraction recovery (%)		Matrix effect (%)	
		Mean ± SD	RSD%	Mean ± SD	RSD%
Iopromide	20.0	102.69 ± 4.22	4.11	104.08 ± 3.95	3.79
	80.0	99.77 ± 3.23	3.24	93.24 ± 4.89	5.24
	320.0	91.39 ± 1.56	1.71	97.64 ± 3.66	3.74

Iodixanol	20.0	97.68 ± 1.46	1.49	96.64 ± 4.83	4.99
	80.0	100.14 ± 3.06	3.05	90.10 ± 2.96	3.29
	320.0	99.43 ± 4.31	4.34	87.88 ± 2.06	2.35

#### 3.2.5. Stability

In this experiment, three concentration levels of QC samples of iopromide and iodixanol were examined for autosampler stability (24 h), room temperature stability (25°C, 4 h), freeze‐thaw stability (three cycles), and long‐term stability (−80°C, 30 days). The RE% were all within −14.6∼13.9% (−15.2∼7.5% for LLOQ), and the RSD% were less than 7.0% (less than 13.2% for LLOQ). The data are shown in Tables 6 and 7.

**Table 6:** 24‐h autosampler stability of iopromide and iodixanol in human plasma (*n* = 3).

Compound	Nominal concentration (μg/mL)	0 h			12 h			24 h		
		Mean ± SD	RSD%	RE%	Mean ± SD	RSD%	RE%	Mean ± SD	RSD%	RE%
Iopromide	20.0	18.49 ± 0.29	1.60	7.50	19.46 ± 0.14	0.75	−2.70	20.46 ± 0.65	3.19	2.29
	80.0	67.24 ± 1.86	2.80	13.9	64.48 ± 0.42	0.65	−14.40	70.56 ± 0.66	0.93	−11.80
	320.0	301.08 ± 3.68	1.22	5.90	300.51 ± 3.05	1.02	−6.00	326.15 ± 2.68	0.82	1.90

Iodixanol	20.0	19.61 ± 1.55	7.90	−1.90	17.49 ± 0.21	1.20	−12.5	17.93 ± 0.86	4.80	−10.3
	80.0	83.23 ± 2.60	3.10	4.00	79.00 ± 2.14	2.70	−1.20	80.62 ± 5.64	7.00	0.70
	320.0	349.04 ± 3.83	1.10	9.00	360.17 ± 12.80	3.60	12.5	351.47 ± 8.72	2.50	9.80

**Table 7:** Examination of room temperature placement, frozen and thaw, and long‐term stability of iopromide and iodixanol.

Compound	Nominal concentration (μg/mL)	Bench‐top (4 h)			Freeze‐thaw			Long‐term stability (30 days)		
		Mean ± SD	RSD%	RE%	Mean ± SD	RSD%	RE%	Mean ± SD	RSD%	RE%
Iopromide	20.0	18.81 ± 0.26	1.40	−5.90	20.76 ± 0.48	2.30	3.80	20.58 ± 0.21	1.00	2.80
	80.0	67.51 ± 1.11	1.70	−14.6	72.40 ± 0.80	1.10	−9.40	72.72 ± 0.93	1.30	−9.00
	320.0	334.53 ± 3.09	0.90	4.50	346.45 ± 7.80	2.30	8.20	351.80 ± 8.59	2.40	9.90

Iodixanol	20.0	16.96 ± 0.71	4.19	−15.2	21.61 ± 1.41	6.50	8.00	19.69 ± 3.19	13.21	−1.50
	80.0	72.51 ± 2.51	3.46	−9.40	77.82 ± 11.16	14.3	−2.70	90.87 ± 2.18	2.40	13.5
	320.0	321.70 ± 19.29	6.00	0.53	291.88 ± 11.63	4.00	−8.70	344.56 ± 16.01	4.65	7.60

#### 3.2.6. Dilution Effect

To evaluate the dilution effect, spiked samples of iopromide and iodixanol were prepared at a concentration of 3000.0 μg/mL and subsequently diluted ten‐fold with blank plasma (resulting in a final concentration of 300.0 μg/mL, within the calibration range). Five replicate samples of the diluted solution were prepared and analyzed. The results showed RSD% of 4.4% for iopromide and 4.8% for iodixanol, and RE% of −4.1% for iopromide and 2.1% for iodixanol, respectively. Since the RSD% was less than 15% and the RE% was within ±15%, the dilution effect observed in this study met the methodological requirements for bioanalytical validation.

### 3.3. Application of the Method

**Figure 3:**
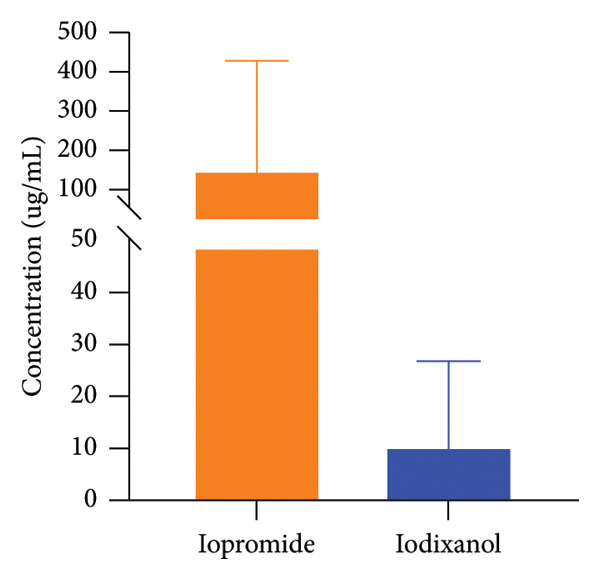
Concentration distribution of iopromide and iodixanol in human plasma.

The method was successfully applied to quantify the concentration levels of both iopromide and iodixanol in patient plasma samples. This experimental protocol has been approved by the Ethics Committee of Shanghai Changzheng Hospital (Approval No. [2023‐025]), and all participants read and signed informed consent forms. A total of 241 plasma samples were collected from patients, comprising 216 samples containing iopromide and 29 samples containing iodixanol. The sampling time was set at the early morning of the next day (approximately 24 h after drug administration). It was noted that only one out of the 216 patients receiving iopromide exhibited a plasma concentration below the LLOQ. In addition, the plasma concentrations of iopromide in 13 patients exceeded the upper limit of quantitation, with the highest recorded concentration reaching 3027.26 μg/mL. As shown in Figure 3, plasma levels of iodixanol are significantly lower than those of iopromide; among the 29 samples, 17 had concentrations below the LLOQ for iodixanol, whereas the highest concentration reached 57.92 μg/mL.

The pronounced disparities in systemic exposure likely stem from fundamental pharmacokinetic and dosing differences between the low‐osmolar agent iopromide and the iso‐osmolar agent iodixanol, which may consequently affect their safety and tolerability. The notably lower exposure observed with iodixanol could correlate with a diminished risk of adverse effects, such as nephrotoxicity or allergic reactions, although further validation remains necessary [18]. These findings highlight the clinical importance of monitoring exposure in an agent‐specific manner, especially among vulnerable patient groups. Future studies should aim to enlarge the iodixanol cohort and directly associate exposure levels with toxicity outcomes to better inform contrast agent selection and individualized dosing, promoting the safe application of CM.

## 4. Conclusions

This study developed a sensitive, accurate, and rapid LC‐MS/MS method for the simultaneous quantification of two iodinated contrast agents (iopromide and iodixanol). For plasma pretreatment, this method employed protein precipitation, which achieved consistent high extraction recoveries and minimal matrix effects (87.88%–104.08% for both analytes). The method has been verified to be suitable for clinical high‐throughput analysis of these contrast agents in patient plasma. Significant individual variations in plasma exposure concentrations of the two contrast agents were observed among different patients. These findings may prove valuable for future clinical assessments regarding the relationship between contrast agent concentrations and the risk of contrast‐associated kidney injury or other adverse reactions in patients.

## Conflicts of Interest

The authors declare no conflicts of interest.

## Author Contributions

Liya Ye: methodology, formal analysis, data curation, investigation, and writing–original draft. Nianxi Yu: investigation, writing–original draft, and writing–review and editing. Peng Huan: investigation, writing–original draft, and writing–review and editing. Zhengyan Liang: methodology, formal analysis, and investigation. Zhenhui Jiang: investigation, writing–original draft, and writing–review and editing. Xinran Wang: investigation and writing–review and editing. Zhipeng Wang: conceptualization, validation, investigation, writing–original draft, and writing–review and editing. Xia Tao: conceptualization, methodology, validation, and formal analysis. Shouhong Gao: investigation, data curation, writing–original draft, writing–review and editing, visualization, and supervision. Liya Ye, Nianxi Yu, and Peng Huan contributed equally to this work.

## Funding

This study was supported by the National Natural Science Foundation of China (82204819, 82274173), the Leading Project of Oriental Talent Program of Shanghai (SHSLJRC‐TX), and the Shenlan Talent Project of Naval Medical University (SL63).

## Data Availability

The data that support the findings of this study are available from the corresponding authors upon reasonable request.
